# Stereotactic Body Radiation Therapy as an Alternative Treatment for Small Hepatocellular Carcinoma

**DOI:** 10.1371/journal.pone.0079854

**Published:** 2013-11-08

**Authors:** Sang Min Yoon, Young-Suk Lim, Mee Jin Park, So Yeon Kim, Byungchul Cho, Ju Hyun Shim, Kang Mo Kim, Han Chu Lee, Young-Hwa Chung, Yung Sang Lee, Sung Gyu Lee, Yu Sun Lee, Jin-hong Park, Jong Hoon Kim

**Affiliations:** 1 Department of Radiation Oncology, Asan Medical Center, University of Ulsan College of Medicine, Songpa-gu, Seoul, Republic of Korea; 2 Department of Gastroenterology, Asan Medical Center, University of Ulsan College of Medicine, Songpa-gu, Seoul, Republic of Korea; 3 Department of Radiology, Asan Medical Center, University of Ulsan College of Medicine, Songpa-gu, Seoul, Republic of Korea; 4 Department of Surgery, Asan Medical Center, University of Ulsan College of Medicine, Songpa-gu, Seoul, Republic of Korea; Osaka University Graduate School of Medicine, Japan

## Abstract

**Background:**

Even with early stage hepatocellular carcinoma (HCC), patients are often ineligible for surgical resection, transplantation, or local ablation due to advanced cirrhosis, donor shortage, or difficult location. Stereotactic body radiation therapy (SBRT) has been established as a standard treatment option for patients with stage I lung cancer, who are not eligible for surgery, and may be a promising alternative treatment for patients with small HCC who are not eligible for curative treatment.

**Materials and Methods:**

A registry database of 93 patients who were treated with SBRT for HCC between 2007 and 2009 was analyzed. A dose of 10-20 Gy per fraction was given over 3-4 consecutive days, resulting in a total dose of 30-60 Gy. The tumor response was determined using dynamic computed tomography or magnetic resonance imaging, which was performed 3 months after completion of SBRT.

**Results:**

The median follow-up period was 25.6 months. Median size of tumors was 2 cm (range: 1-6 cm). Overall patients’ survival rates at 1 and 3 years were 86.0% and 53.8%, respectively. Complete and partial tumor response were achieved in 15.5% and 45.7% of patients, respectively. Local recurrence-free survival rate was 92.1% at 3 years. Most local failures were found in patients with HCCs > 3 cm, and local control rate at 3 years was 76.3% in patients with HCC > 3 cm, 93.3% in patients with tumors between 2.1-3 cm, and 100% in patients with tumors ≤ 2 cm, respectively. Out-of-field intrahepatic recurrence-free survival rates at 1 and 3 years were 51.9% and 32.4%, respectively. Grade ≥ 3 hepatic toxicity was observed in 6 (6.5%).

**Conclusions:**

SBRT was effective in local control of small HCC. SBRT may be a promising alternative treatment for patients with small HCC which is unsuitable for other curative therapy.

## Introduction

Hepatocellular carcinoma (HCC) is the third most common cause of death from cancer worldwide [1]. Current practice guidelines recommend hepatic resection, liver transplantation, and percutaneous ablation as curative treatment options [2]. However, hepatic resection can only be offered to 10% to 30% of patients at diagnosis because of various clinical reasons [3], and the use of liver transplantation is also very limited due to the lack of donors and stringent indications. Although percutaneous ablative therapy, including radiofrequency ablation (RFA) and percutaneous ethanol injection (PEI), can also be used with curative intent for the treatment of small HCC which is unsuitable for surgery, this ablative therapy cannot be safely performed when HCC lesions are positioned at deep locations, near to the bile duct or large vessel, at the top of the dome, or are undetectable by ultrasound. Therefore, alternative non-invasive local therapeutic modalities are indispensable in these clinical settings.

Recent improvements in radiotherapy techniques, including the conformal delivery of radiation, techniques that account for respiratory motion, image-guided radiotherapy (IGRT), and information on partial volume liver tolerance, allow the delivery of higher doses of radiation to these tumors than previously thought possible, thereby allowing radiotherapy to be used as an alternative option for treating HCC [4]. Of the available radiotherapy options, stereotactic body radiation therapy (SBRT) has emerged as a non-invasive local treatment option for patients with HCC when established curative treatment modalities cannot be applied. Although prospective studies remain sparse at this time, many previous clinical studies have reported SBRT to be safe and efficacious for the treatment of HCC [5-8]. 

In our current study, we report our clinical experiences with SBRT as an alternative treatment for small, unresectable HCC and evaluate the long-term efficacy and safety of this highly sophisticated treatment modality.

## Materials and Methods

### Ethics statement

This study was approved by the Institutional Review Board of the Asan Medical Center, and informed consent in writing was obtained from each patient in the study.

### Patients

Patients who were treated with SBRT for primary or recurrent HCC were registered and the database was retrospectively reviewed between March 2007 and December 2009. Eligibility criteria for the present study included the following: (1) the HCC lesion was not suitable for surgery due to liver cirrhosis, no sufficient remnant liver for resection after previous surgery, and patients’ refusal of surgery; (2) the HCC was located in liver surface, near to the bile duct or large vessels, or at the top of the dome where percutaneous ablative therapies cannot be safely performed; (3) the HCC was confined to the liver without extrahepatic metastases; (4) the HCC was < 6 cm across its longest diameter, and ≤ 3 lesions were present; (5) the HCC demonstrated no evidence of major vascular invasions; (6) liver function was classified as Child-Pugh class A or B; (7) an adequate residual functional liver volume was evident (> 700 cc); (8) there was a sufficient distance (> 2 cm) between the HCC and adjacent organs at risk, such as the duodenum, stomach, colon, and spinal cord; (9) an incomplete response after transarterial chemoembolization (TACE) or unsuitable for TACE due to the lesion non-visibility on hepatic angiogram; and (10) no prior history of external beam radiotherapy.

In all of our patients, the diagnosis of HCC was based on (1) histological confirmation; or (2) a characteristic tumor appearance by at least two imaging studies (including dynamic computed tomography (CT) scans, dynamic enhanced magnetic resonance imaging (MRI) scans and angiograms); and (3) the presence of risk factors, including hepatitis B virus, hepatitis C virus infection and cirrhosis. 

### Simulation and target volume delineations

At least one week before CT simulation, we implanted three gold seeds (CIVCO Medical Solutions, Kalona, IA, USA) into the liver parenchyma around the tumor under sonographic guidance in almost every patient. The gold seeds were not implanted in some patients who had surgical clips or compact iodized oil, which still remained after previous treatments. All patients were immobilized in the supine position using a vacuum cushion, and while freely breathing 4-dimensional (4D) CT scanning was performed using 16-slice CT system (GE LightSpeed RT 16; GE Healthcare, Waukesha, WI, USA). A Real-time Position Management respiratory gating system was used to record the patients’ breathing patterns (Varian Medical Systems, Palo Alto, CA, USA). All CT datasets were sorted into 10 phase bins that corresponded to the respiratory phase using 4D imaging software (Advantage 4D; GE Healthcare). 

The gross tumor volume (GTV), as determined by dynamic enhanced CT or MRI, included an enhanced mass at the end-expiratory phase of the CT image (50% phase). No GTV to clinical target volume margin was added for taking into account subclinical extension. The internal target volume (ITV) was delineated as the sum of the individual GTVs, as defined within the gated phases of respiration (usually 30-70% phase). The planning target volume (PTV) was expanded to include a 0.5-cm margin from the ITV. The whole and normal liver, both kidneys, spinal cord, duodenum, and stomach were delineated and 3- dimensionally reconstructed.

### Treatment planning and delivery

SBRT planning was performed using a 3-dimensional radiotherapy planning system (Eclipse; Varian Medical Systems) that used multiple static conformal beams with energies of 6- or 15-MV photons. A dose of 10-20 Gy (median: 15 Gy) per fraction was given over 3-4 consecutive days, resulting in a total dose of 30-60 Gy (median: 45 Gy) being administered to the isodose line covering the PTV. The total dose was mainly determined based on general dosing guidelines after determining the dose to be administered to the normal liver, including the following: (1) the maximum dose allowed to 700 cc of normal liver was estimated to be 15 Gy in three fractions: and (2) the mean dose administered to normal liver was not to exceed 13 Gy in three fractions [9,10]. Dose limitations to other critical structures included the following: 2 cc of the esophagus or large bowel were to be limited to a total dose of < 21 Gy, 2 cc of the stomach or duodenum were to be limited to a total dose of < 18 Gy, and 2 cc of the spinal cord were to be limited to a total dose of 18 Gy.

Image guidance was performed in two stages before administering each fraction of SBRT using On-Board Imager (Varian Medical Systems). First, cone-beam CT was done and 3D matching was performed. Second, gated fluoroscopy was performed in the anterior-posterior and lateral directions to confirm the marker positions at the end-exhale phase.

### Evaluation and statistics

All patients were examined during SBRT to assess acute toxicity. After treatment, regular follow-up examinations were performed at 2-3 month intervals. A review of each patient’s prior medical history, physical examinations, complete blood counts, biochemical profiles, tumor markers, and imaging studies were performed at each follow-up. Adverse effects related to SBRT were graded according to the Common Terminology Criteria for Adverse Events (CTCAE; version 3.0). Radiation-induced hepatic toxicity was also graded according to CTCAE or any decline in liver function using Child-Pugh score in the absence of documented progressive disease within three months after SBRT.

Tumor response was defined at three months after the completion of SBRT according to both the Response Evaluation Criteria in Solid Tumors criteria (RECIST version 1.1) and the modified RECIST with the consensus of two radiologists (KSY and PMJ). Local failure was defined as the recurrence of the treated lesion, intrahepatic recurrence was defined as recurrence within the liver outside the treated lesion, and extrahepatic metastasis was defined as recurrent disease at any site outside the liver. For local recurrence, the presence of wash-out on the portal and delayed phase images or increase in volume within the irradiated hepatic parenchyma was considered as such.

Overall and recurrence-free survivals were estimated from the date of the start of SBRT to the date of death, the last follow-up examination, or to the date of tumor recurrence, respectively. The probability of cumulative survival was calculated using the Kaplan-Meier method. Correlation between tumor sizes and radiation doses was performed using Spearman correlation analysis. In addition, the existence of a variable-effect relationship was confirmed by logistic regression analysis. These analyses were performed using SPSS (version 12.0; SPSS Inc., Chicago, IL, USA).

## Results

### Patient characteristics

**Figure 1:**
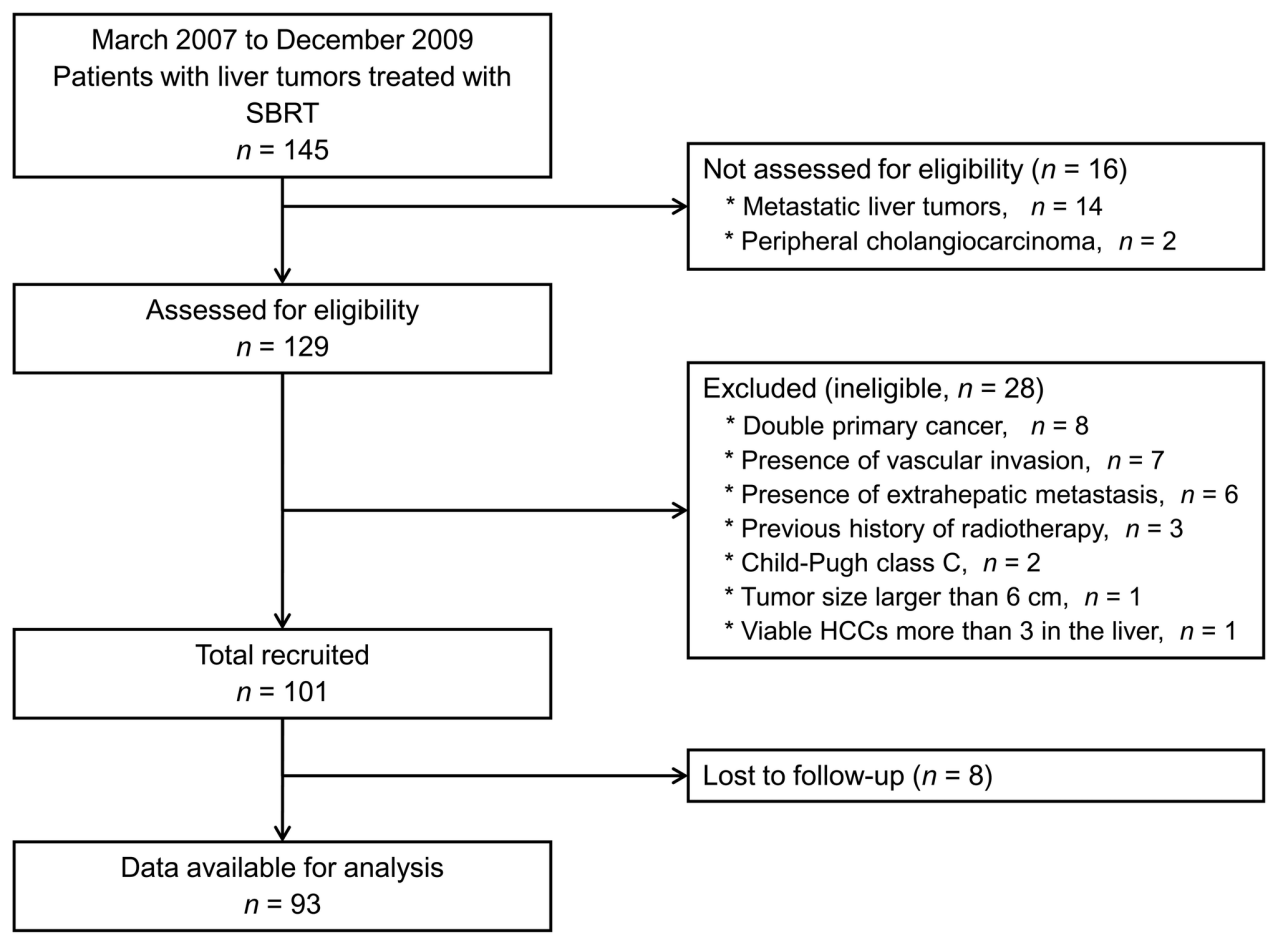
Flow diagram of this study.

A total of 129 patients with HCC who were treated with SBRT were registered between March 2007 and December 2009 at our institution. Among these, 28 patients were not included in our current analysis for the various reasons in Figure 1. The remaining 93 patients (103 lesions) met all of the enrollment criteria and were included in this analysis (Table 1). The study population was mostly male (80.6%), demonstrating a median age of 61 years (range: 42-86 years). Sixty-nine patients had liver function of Child-Pugh class A, and median tumor size was 2 cm. Eighty-nine (95.7%) patients were identified to have liver cirrhosis. Only 1 patient was treatment-naïve, and all other patients had received various courses of previous therapies, including hepatic resection, TACE, RFA, or PEI before receiving SBRT. However, additional locoregional treatments were not performed on recurrent or residual viable HCCs if salvage SBRT was considered for the lesion. Before CT simulation, 88 patients (94.6%) were implanted with 3 gold seeds to serve as fiducial markers for IGRT.

**Table 1:** Patient characteristics.

Variables	No. of patients (%)
Gender	
Male	75 (80.6)
Female	18 (19.4)
Age (years)	
Median	61
Range	42-86
Child-Pugh class	
A	69 (74.2)
B	24 (25.8)
Viral etiology	
HBV	69 (74.2)
HCV	12 (12.9)
Others	12 (12.9)
Liver cirrhosis	
Yes	89 (95.7)
No	4 (4.3)
Tumor size^*^	
1.0-2.0 cm	54 (52.4)
2.1-3.0 cm	18 (17.5)
3.1-4.0 cm	22 (21.4)
4.1-5.1 cm	6 (5.8)
5.1-6.0 cm	3 (2.9)
Number of viable tumors before SBRT	
1	83 (89.2)
2	10 (10.8)
Alpha-fetoprotein (ng/mL)	
Range	0.8-2490
≤ 200	78 (83.9)
> 200	15 (16.1)
Prior treatments	
None	1 (1.1)
TACE only	48 (51.6)
TACE, RFA	21 (22.6)
TACE, PEI	4 (4.3)
TACE, RFA, PEI	2 (2.1)
Resection	1 (1.1)
Resection, TACE	11 (11.9)
Resection, TACE, RFA	2 (2.1)
Resection, TACE, PEI	1 (1.1)
RFA	2 (2.1)

Abbreviations: HBV, hepatitis B virus; HCV, hepatitis C virus; SBRT, stereotactic body radiation therapy; TACE, transarterial chemoembolization; RFA, radiofrequency ablation; PEI, percutaneous ethanol injection.

* One hundred and three tumors were analyzed for the size of tumors.

### Overall survival and tumor response

**Figure 2:**
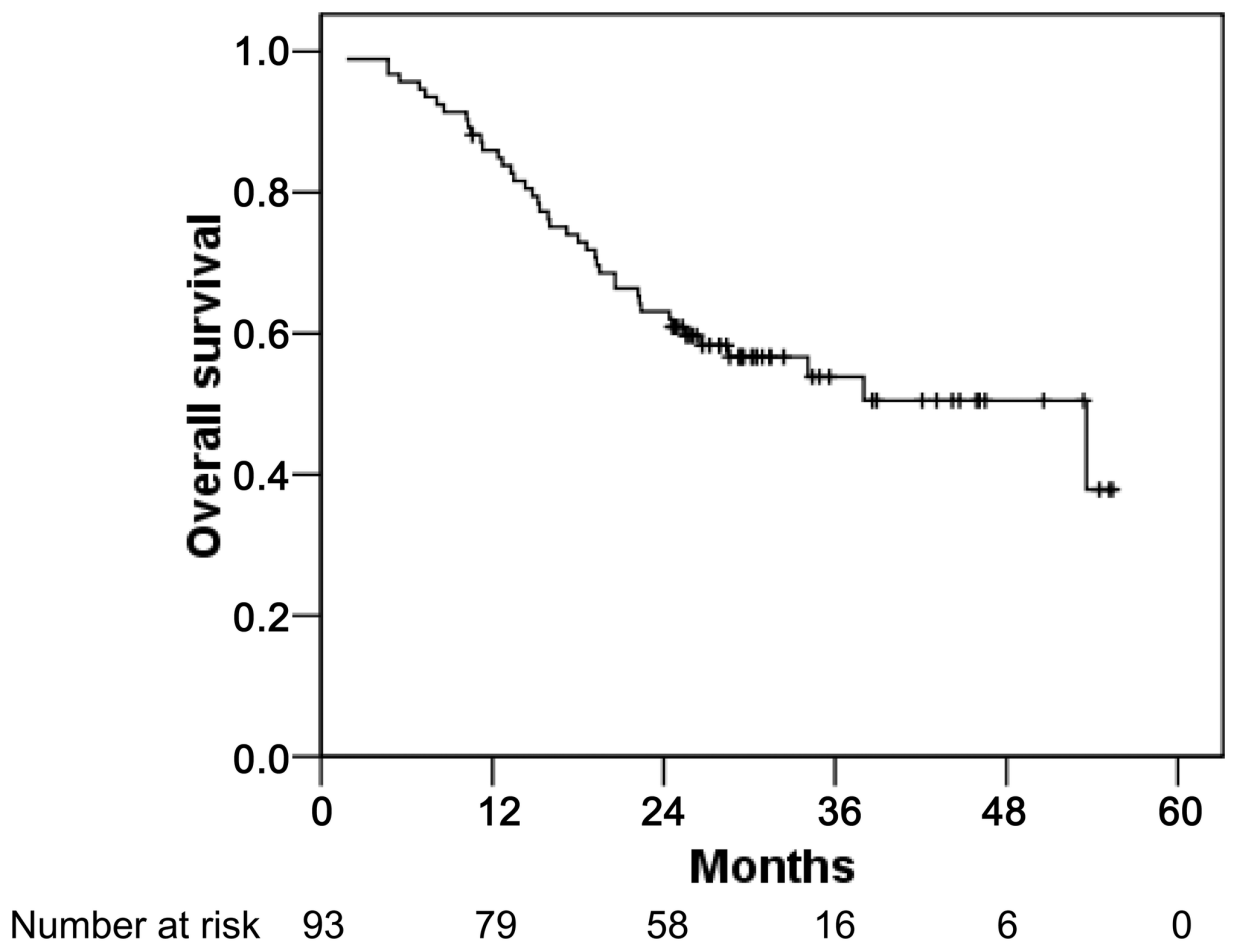
Overall survival rates of the enrolled patients. The 1 and 3 years survival rates are 86.0% and 53.8%, respectively.

The median follow-up period for all patients was 25.6 months (range: 1.8–55.4 months). At the time of analysis, 51 patients were alive and 42 patients were deceased. The causes of death were as follows: intrahepatic or extrahepatic tumor progression in 29, progression of liver cirrhosis in 8, other comorbidity in 2, complications after the following treatment in 1, and unknown cause(s) in 2. The 1 and 3 years overall survival rates were 86.0% and 53.8%, respectively (Figure 2).

CT or MRI at 3 months after SBRT was evaluated in 91 patients (101 lesions). Of these, 16 (15.5%) achieved complete response, 47 (45.7%) achieved partial response, and 38 (36.9%) achieved stable disease, yielding an objective response rate of 61.2% according to the RECIST criteria (Table 2). Using the modified RECIST criteria, however, 53 (51.5%) achieved complete response, 22 (21.4%) achieved partial response, and 26 (25.2%) achieved stable disease, yielding an objective response rate of 72.9% (Table 2). No progressive disease was observed in the treated HCCs.

**Table 2:** Response rates at 3 months after stereotactic body radiation therapy.

	RECIST (version 1.1)	Modified RECIST
	No. of lesions (%)	No. of lesions (%)
Complete response	16 (15.5)	53 (51.5)
Partial response	47 (45.7)	22 (21.4)
Stable disease	38 (36.9)	26 (25.2)
Not evaluated	2 (1.9)	2 (1.9)
Response rate (CR+PR)	63 (61.2)	75 (72.9)

Abbreviations: RECIST, Response Evaluation Criteria in Solid Tumors; CR, complete response; PR, partial response.

### Patterns of failure and recurrence after SBRT

**Figure 3:**
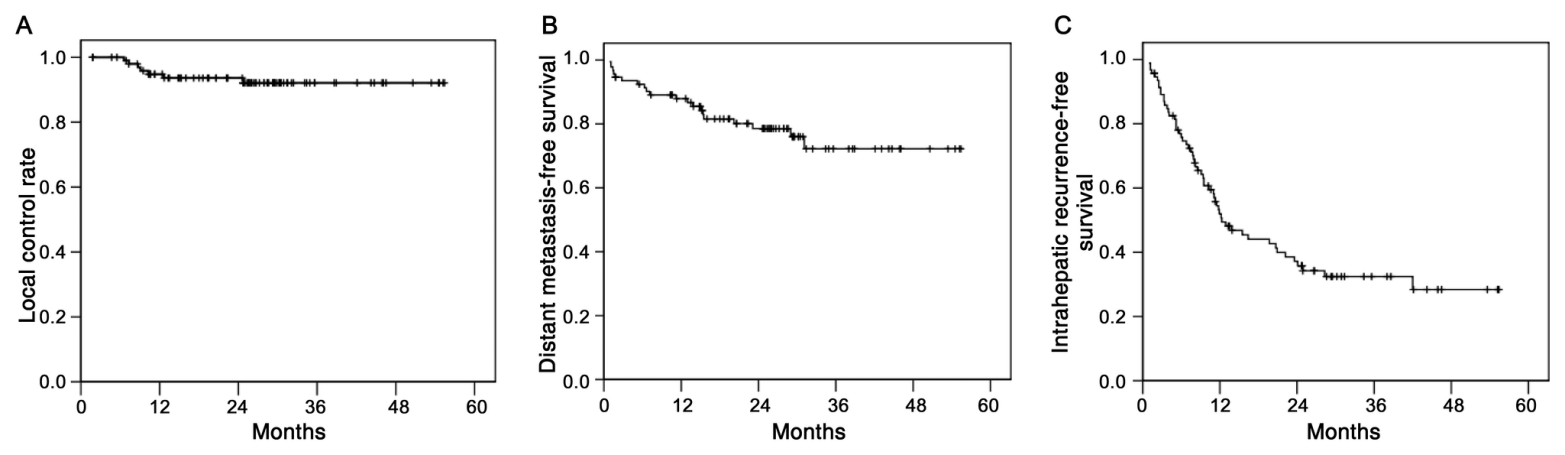
Local control and recurrence-free survival rates. (A) The local control rates at 1 and 3 years were 94.8% and 92.1%, respectively. (B) Distant metastasis-free survival rates and (C) Intrahepatic recurrence-free survival rates following SBRT.

Seven local failures were recorded at the time of analysis. Intrahepatic (i.e., out-of-field) recurrence was the main cause of failure (58 of 92 patients), and distant metastasis developed in 20 patients during the follow-up periods. Local control rates at 1 and 3 years were 94.8% and 92.1%, respectively (Figure 3A), and distant metastasis-free survival rates at 1 and 3 years were 87.9% and 72.2%, respectively (Figure 3B). However, intrahepatic recurrence-free survival rates at 1 and 3 years were 51.9% and 32.4%, respectively (Figure 3C)

**Figure 4:**
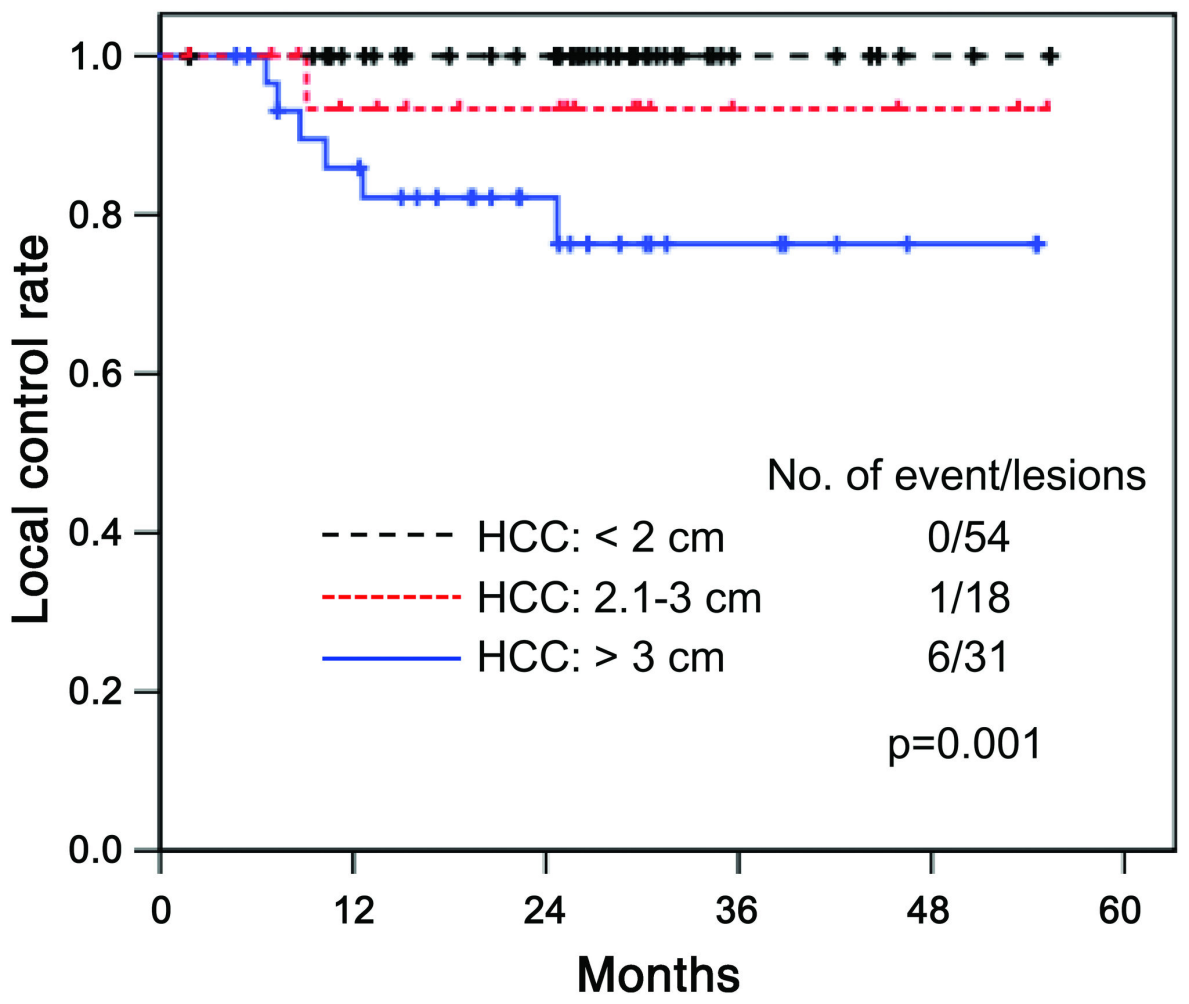
Analysis of local failures. Most local failures occurred in patients with HCCs > 3 cm, and the local control rate at 3 years was 76.3% in these cases, 93.3% in patients with HCCs between 2.1-3 cm, and 100% in patients with HCCs < 2 cm, respectively (p=0.001).

The incidence of local failure was significantly related to tumor size. Most local failures were found in patients with HCCs > 3 cm, and local control rate at 3 years was 76.3% in patients with HCC > 3 cm, 93.3% in patients with tumors between 2.1-3 cm, and 100% in patients with tumors ≤ 2 cm, respectively (p=0.001) (Figure 4). There was no significant correlation between tumor sizes and radiation doses (p=0.078). Logistic regression analysis also revealed that the tumor size was the only significant factor determining the local tumor control (p=0.001). Other variables, such as the age, Child-Pugh score, pretreatment alpha-fetoprotein level, and radiation dose were not significant factors.

### Additional treatment after SBRT for New Recurrent Lesions

After the diagnosis of new recurrences, 65 patients received various types of additional treatments, including locoregional treatments for intrahepatic recurrences (e.g., segmentectomy, TACE, PEI, RFA, SBRT) or treatments for distant metastases (e.g., sorafenib, metastatectomy, SBRT for metastatic lesions, palliative external beam radiotherapy). Liver transplantation was performed in 7 patients. Of these, 4 patients received transplantation for the treatment of liver cirrhosis without tumor recurrence and 3 patients received transplantation in combination with other loco-regional treatments after intrahepatic recurrence.

### Treatment-related toxicity

Symptomatic complications, including septic shock, due to the insertion of the gold seeds occurred in 1 patient. However, this individual recovered well before the start of SBRT with supportive care. All patients received the planned SBRT regimen with no interruptions due to intolerable side effects. Fatigue and anorexia were the most common acute toxicities, but these were mostly CTCAE grade 1. Grade ≥ 3 hepatic toxicities, without progression of intrahepatic HCC, which may have been related to radiation, were observed in 6 (6.5%) patients within 3 months of SBRT and are summarized in Table 3. The main patterns of hepatic toxicities were elevations in serum liver enzyme levels or bilirubin levels. Nine (9.7%) patients also experienced an elevation in the Child-Pugh score to > 2 within 3 months of SBRT. Of these, one patient, who experienced grade 4 hyperbilirubinemia, died due to hepatic failure at 2 months after the completion of SBRT. However, it was difficult to differentiate the cause of hepatic failure from SBRT or other unrevealed reasons, because the treated tumor was small (1.5 cm), also located in the liver dome area, and the liver function was good (Child-Pugh class A). The remainder of the patients showed improved or stable liver function during the follow-up period. Both the pretreatment Child-Pugh class and the radiation dose were not correlated with the grade ≥ 3 hepatic toxicities in logistic regression analyses (p=0.081 and p=0.153, respectively). There was no gastrointestinal complication such as bleeding or perforation, during the follow-up periods. Two patients developed rib fractures that did not require any specific treatment at 12 months and 18 months after SBRT, respectively. In these cases, the PTV included a portion of the fractured ribs. The total dose to those ribs was 30 Gy and 45 Gy, in three fractions, respectively. Biliary stricture developed in 1 patient, whose tumor was located in the central liver (segment 4), at 26 months after SBRT.

**Table 3:** Summary of the labortory finding and the radiation dose in patients who experienced the grade ≥ 3 hepatic toxicities.

Case No.	CTCAE grade	Baseline liver function before SBRT				Dose (Gy)	BED(Gy_10_)	Hightest level after SBRT (≤ 3 months)			Normalized or stabilized
		C-P class	AST (IU/L)	ALT(IU/L)	t-Bilirubin (mg /dL)			AST (IU/L)	ALT(IU/L)	t-Bilirubin (mg /dL)	
1	3	7	57	34	2.9	45	112.5	66	36	5.4	Yes
2	3	6	105	146	1.8	36	79.2	276	298	1.4	Yes
3	3	5	57	116	1.4	36	79.2	70	216	1.2	Yes
4	3	7	29	15	1.1	45	112.5	230	58	2.5	Yes
5	4	7	78	19	6.2	40	80	696	493	8.9	Yes
6	5	5	37	33	1.5	45	112.5	390	547	41.4	No

Abbreviations: CTCAE, the Common Terminology Criteria for Adverse Events; SBRT, Stereotactic Body Radiation Therapy; C-P class, Child-Pugh class; AST, aspartate aminotransferase; ALT, alanine aminotransferase; t-Bilirubin, total bilirubin; BED, biologically effective dose.

## Discussion

Hepatic resection is the primary curative treatment for HCC. Due to advances in surgical techniques and improvements in postoperative management, the 5-year survival rate has increased to about 70%, especially for small HCCs < 5 cm in diameter [11]. Unfortunately, only a small proportion of patients with HCC can undergo hepatic resection. For patients with early-stage HCC who are not suitable for surgery, percutaneous ablative therapy is another curative treatment option. Although, randomized trials comparing hepatic resection and RFA for the treatment of HCC reach different conclusions [12-14], RFA can achieve satisfactory local control rates and similar survival outcomes compared with hepatic resection, demonstrating 5-year survival rates of 50-75% in patients with liver function that is classified as Child-Pugh class A [15]. Based on these clinical outcomes, the recently updated Barcelona Clinic Liver Cancer guideline recommended ablative therapy as the curative treatment option for very early-stage HCC [16]. However, in some cases RFA cannot be safely and effectively performed, just like surgical resection. Therefore, there is an opportunity for SBRT to be performed on these selected HCC cases.

Here, we reported a very high local recurrence-free survival rate of 92.1% at 3 years. Many previous studies have reported 2 or 3 years local progression-free survival rates that range from 66.4% to 90% [5,6,17-19]. Although, these results cannot be directly compared due to the different inclusion criteria and prescribed doses, our results show that tumor ablation is possible using high dose of radiation and precision radiotherapy techniques. We assume that thorough IGRT is one of the most important ways to obtain a high local control rate over a long follow-up duration. In our series, most of patients were implanted with 3 gold seeds and pretreatment verification of the marker positions was conducted using 2 separate processes before the administration of each fraction to each patient. Even though pretreatment verifications are eagerly performed, there are still various uncertainties regarding to the respiration during treatment time. In consideration of our lower cumulative incidence of local failure, our PTV setup margin can cover the intrafractional respiratory uncertainties. In addition, a high local control rate may be associated with the inclusion of relatively small-sized tumors in this study.

In the present study cohort, the incidence of local recurrence was significantly related to tumor size. Although, there were some different size criteria compared with previously reported results for SBRT, other authors have also reported a relationship between tumor size and the local control rate. Kwon et al. reported that patients with a tumor volume < 32 cc demonstrate better in-field tumor responses and in-field progression-free survival rates than those with tumor volumes ≥ 32 cc [6]. Andolino et al. have reported a local control rate at 2 years of 90%, exceeding the local control rate of 65% at 1 year that was reported by Tse et al. They explained that the higher median total dose and higher median tumor volume contributed to the differences in the local control rates [5,8]. In other RFA series, very similar findings were observed, indicating that the local tumor control rate decreases as the tumor size increases [20,21]. All of these findings may be related to the prognostic significance of small-size HCCs. In a previous surgical series, it was revealed that large tumor size is a biological predictor of poor clinical prognosis, demonstrating a higher incidence of occult vascular invasion and advanced histological grade [22]. Therefore, care must be taken when treating HCCs > 3 cm, and prospective studies are needed to clarify the relationship between radiation dose escalation, target volume expansion, and the control of microscopic invasion. However, most HCC patients suffer from chronic liver disease, including liver cirrhosis, and have a limited functional normal liver volume. This may be an obstacle to performing studies on dose escalation because of the risks of hepatic toxicity following high-dose radiotherapy.

In our present analysis, the 3-year survival rate was 53.8% for patients with small, recurrent HCC. This result is comparable or slightly higher survival rates than that of other previously reported studies on SBRT [5,6,17,19]. Because SBRT was used as a salvage treatment following various locoregional therapies in most previously reported studies, it is difficult to compare the survival outcomes of SBRT, resection, and RFA when they are used as the initial treatment modalities. In the case of recurrent HCC following hepatectomy, repeated resection demonstrates slightly better survival outcomes than RFA, and the results of RFA are comparable to those of SBRT [23]. To the best of our knowledge, no standard treatment has been established for recurrent HCC; SBRT can be a good treatment option for patients with recurrent HCC and can achieve comparable survival outcomes to other aggressive salvage treatment options.

The primary failure pattern was intrahepatic recurrence and the 3-year intrahepatic recurrence-free survival rate was only 32.4%. Although HCC recurrence rates vary across earlier studies, recurrence outside the radiation field is the main cause of failure in previously published reports on SBRT [5,6,17]. Recently, a very similar rate of intrahepatic recurrence was reported following the administration of salvage hypofractionated radiotherapy to patients with recurrent small HCC [24]. The authors suggested that intrahepatic recurrence might occur because patients received salvage radiotherapy at a more advanced disease state even if the recurrent tumor was small [24]. In our present study, most patients, except 1 individual who received SBRT as the initial treatment modality, were also treated with a median number of 5 courses (range 1-18) of locoregional therapies before SBRT, including resection, TACE, PEI, or RFA. Therefore, particular attention should be paid to interpreting the intrahepatic recurrence rates between studies. Moreover, in order to overcome the limitations of these clinical settings, trials are needed that assess the efficacy of various combinations of SBRT and other systemic treatment modalities including sorafenib.

Grade ≥ 3 hepatic toxicities were observed in 6.5% of patients within 3 months after the SBRT and most patients showed improved or stable liver function without a serious hepatic damage during the follow-up period. This incidence may be acceptable, compared with that of the previous studies (range, 0-25.8%) [5-8,17,18]. Among the patients who experienced the hepatic toxicities, one patient died due to the hepatic failure at 2 months after the treatment similar to those reported by Mendez Romero et al. [7]. However, the cause of this hepatic failure is difficult to pin down precisely. The hepatic function may be affected by various causes in patients with chronic liver disease, as we are all well aware, this event may be quite difficult to explain as what the real cause is. Although the risk factors for the hepatic damage were not found in the present study, the pretreatment Child-Pugh class is one of the important factors in predicting the hepatic toxicities according to the previous reports. Therefore, more attention could be given in prescribing the total dose for patients with Child-Pugh B cirrhosis [5,7,25]. Moreover, additional efforts aimed at finding the clinical and dose-volumetric parameters to predict the risk of hepatic toxicities in a large series of HCC patients treated with SBRT are needed.

In conclusion, the SBRT is a noninvasive and an excellent ablative treatment modality for patients with small, primary/recurrent HCC. Our current results indicate that SBRT can be a good alternative modality for the treatment of small HCCs that are unsuitable for surgical resection or local ablative therapy. In order to accurately determine its efficacy and impact on overall survival compared with other local modalities, well-designed prospective investigations of SBRT are needed. In addition, further trials are also necessary to assess the efficacy of combinations of SBRT and other novel targeted agents for the treatment of patients with recurrent HCC.
